# Tremor evaluation using smartphone accelerometry in standardized settings

**DOI:** 10.3389/fnins.2022.861668

**Published:** 2022-08-01

**Authors:** Gürdal Sahin, Pär Halje, Sena Uzun, Andreas Jakobsson, Per Petersson

**Affiliations:** ^1^Integrative Neurophysiology and Neurotechnology, Department of Experimental Medical Sciences, Lund University, Lund, Sweden; ^2^Department of Internal Medicine, Hässleholm Hospital, Region Skåne, Hässleholm, Sweden; ^3^Skåneuro Neurology Clinic, Lund, Sweden; ^4^Department of Clinical Sciences of Malmö and Lund, Lund University, Lund, Sweden; ^5^Centre for Mathematical Sciences, Mathematical Statistics, Lund University, Lund, Sweden; ^6^Department of Integrative Medical Biology, Umeå University, Umeå, Sweden

**Keywords:** essential tremor, Parkinson’s disease, inertia sensors, neuromodulation, closed-loop

## Abstract

Tremor can be highly incapacitating in everyday life and typically fluctuates depending on motor state, medication status as well as external factors. For tremor patients being treated with deep-brain stimulation (DBS), adapting the intensity and pattern of stimulation according the current needs therefore has the potential to generate better symptomatic relief. We here describe a procedure for how patients independently could perform self-tests in their home to generate sensor data for on-line adjustments of DBS parameters. Importantly, the inertia sensor technology needed exists in any standard smartphone, making the procedure widely accessible. Applying this procedure, we have characterized detailed features of tremor patterns displayed by both Parkinson’s disease and essential tremor patients and directly compared measured data against both clinical ratings (Fahn-Tolosa-Marin) and finger-attached inertia sensors. Our results suggest that smartphone accelerometry, when used in a standardized testing procedure, can provide tremor descriptors that are sufficiently detailed and reliable to be used for closed-loop control of DBS.

## Introduction

Therapeutic neuromodulation, for example deep-brain stimulation (DBS), can effectively ameliorate tremor in neurological conditions such as essential tremor (ET) and Parkinson’s disease (PD). It is thought, however, that significant improvement in treatment efficacy could be achieved if the stimulation protocols were better adapted to the changing unique needs of the patient. For this reason, closed-loop neuromodulatory systems, where sensor data, informing on the current tremor state, is used as feedback control of the stimulation device has been attracting a growing interest. In principle, any electrical closed-loop neuromodulation system contains three main components that jointly determine system characteristics, and which each presents a separate engineering challenge. That is, (1) sensors that can reliably estimate severity and characteristics of symptoms being treated, (2) control algorithm converting sensor signals into appropriate adjustments of the neuromodulatory stimulation patterns, and (3) one or more stimulating devices passing current into the tissue to modulate neuronal activity. Designing an efficient closed-loop neuromodulation system to improve treatment of tremor therefore requires knowledge of both symptom characteristics and of the underlying pathophysiology. During the last three decades, certain advances have been made in this latter respect in ET and PD. When bilateral upper limb action tremor is present in the absence of other neurological signs, the condition is commonly classified as ET (Bhatia et al., 2018). Several studies point to a central origin of ET and it has been hypothesized that synchronized rhythms in brain networks may generate oscillation frequencies that are being transmitted to the muscles. In line with this notion, thalamotomy or DBS of the ventral intermediate nucleus (VIM) of the thalamus is known to result in effective symptomatic treatment (Vaillancourt et al., 2003; Pedrosa et al., 2014; Huss et al., 2015). Consequently, VIM stimulation has been the preferred surgical target for the treatment of ET and is recommended primarily in elderly with medication refractory ET (Dallapiazza et al., 2019). In a similar way, in PD, thalamic neurons have been found to discharge rhythmic bursts at 3–6 Hz that correlate with limb tremor (Lenz et al., 1988) and VIM stimulation is known to be an effective treatment for PD tremor as well (Cury et al., 2017). However, to achieve symptomatic relief covering the broader range of motor complications in PD, including rigidity and bradykinesia, the subthalamic nucleus (STN) is generally considered a preferred target. Interestingly, in PD patients with severe tremor at rest, the thalamic bursting rhythm can be the predominant oscillation frequency also in the STN (Alonso-Frech et al., 2006).

With respect to characterization of symptoms, a rapid development has recently taken place in the use of inertia sensor techniques. In particular, the development of a range of body-worn sensors incorporated in consumer products such as watches and the almost ubiquitous use of smartphones in everyday life has opened-up for many new ways to monitor movement disorders using commercially available products. However, although several studies have shown a good correspondence between accelerometer data and clinical scores (see e.g., Senova et al., 2015; Haubenberger et al., 2016; Longardner et al., 2019; Vescio et al., 2021) only a few technical solutions have yet been developed to a technological readiness level that is approaching clinical requirements (Luis-Martínez et al., 2020). Thus, in this study we have investigated to what extent the accelerometry technology widely available in smartphones could provide sufficiently detailed characterizations of tremor in ET and PD patients to be used in a closed-loop controlled DBS device; under the assumption that the metric created could be implemented as a simple control algorithm to modify stimulation features. In this context, we foresee that a calibration procedure using a standardized setting will be faster and more reliable than on-line monitoring of movements across widely differing behavioral states. Hence, the data analyzed have been recorded during standard neurological assessments of postural tremor that would be trivial for the patients to carry out independently at home on demand. Finally, to assess the potential limitations of smartphone accelerometry data, phone recordings were here directly compared to miniature inertia sensors attached to the index finger.

## Materials and methods

### Subjects

In total, 33 subjects were included in the study. Of these, 17 with ET (12 males, 5 females) with an average age of 68 years (28–89), 9 with PD tremor (5 males, 4 females) with an average age of 75 years (57—79) were recruited. In addition, seven subjects (1 male, 6 females) with an average age of 51 years (21–77) with diabetes mellitus type 2 without any neurological complications were also included in the study as control group (the study was performed during Covid-19 pandemics, which created difficulties to recruit research persons to the control group. We have therefore used patients with diabetes as control group because of practical and safety issues). The diagnosis of ET was confirmed using the neurological examination and the WHIGET diagnostic criteria (Louis and Pullman, 2001), whereas diagnosis of PD was confirmed using MDS clinical diagnostic criteria for Parkinson’s disease (Postuma et al., 2015). Subjects who were diagnosed with other forms of chronic motor system dysfunctions (e.g., previous stroke or tumor with persistent, significant motor impairments), hallucinations, alcoholism, drug addiction, dementia, or were on medications that can cause tremor or motor impairments, were excluded from the study.

This study was approved by the Swedish Ethical Review Authority with a diary number of 2021-00503 and all of the participants included signed written informed consent. The detailed descriptions of participants are listed in Table 1.

**TABLE 1:** Clinical profile of subjects with essential tremor and Parkinson’s disease.

#	Group	Age (years)	Tremor Side	Gender	Disease duration (years)	Rating score (FTM-TRS)	Current treatment
1	ET	89	R	M	6	14	B
2	ET	82	R	F	11	35	B
3	ET	80	R	M	6	16	None
4	ET	79	R	F	4	26	B
5	ET	78	L	F	5	19	B
6	ET	77	L	M	5	7	B
7	ET	73	R	M	8	5	P
8	ET	69	L	F	5	28	B
9	ET	66	R	F	20	13	B
10	ET	60	R	M	30	29	B
11	ET	57	L	M	15	16	B, C
12	ET	52	R	M	20	10	B
13	ET	46	L	M	40	8	B
14	ET	28	L	M	10	18	B
15	ET	71	R	M	47	14	None
16	ET	72	R	M	7	28	B
17	ET	75	L	M	16	20	B, G
18	PD	79	R	M	4	5	L
19	PD	78	R	F	2	6	L
20	PD	76	R	F	3	14	L
21	PD	75	R	M	8	3	L
22	PD	74	L	M	4	9	L
23	PD	74	L	F	8	15	L, R
24	PD	74	L	M	8	6	L
25	PD	75	R	M	16	20	L
26	PD	68	L	F	16	20	A, C, G, L

A, Amantadin; C, Clonazepam; G, Gabapentin; FTM-TRS, Fahn, Tolosa, Marin Tremor Rating Scale; LD, Levodopa; B, Betablocker; P, Primidone; R, Ropinirole.

### Description of standardized testing procedures

Clinical evaluations were performed in Neurology Unit of the Department of Internal Medicine in Hässleholm Hospital, where the patients were first assessed with a routine neurologic examination. Afterward, the clinical characteristics of tremor were evaluated using “Fahn, Tolosa, Marin Tremor Rating Scale” (Fahn et al., 1993).

Tremor recordings were performed by an experienced nurse in accelerometric recordings. The accelerometer was placed onto the index fingers of the tremor-dominant hand, in a position where it was possible to sense flexion-extension and abduction-adduction axes. The right side was chosen in cases of symmetrical tremor and in control subjects. One-minute long accelerometric recordings were obtained in two different positions. Position 1 (P1): Shoulders forward flexed at 90°, extension at the elbow joint and wrist joint with the palm facing downwards. Position 2 (P2): Shoulder abduction at 90°, elbow flexion and wrist extension with palms facing downward. Directly after finishing accelerometric tremor recordings, a smartphone was strapped to the dorsum of hand. The abovementioned protocol was then repeated using the smartphone for tremor recording.

### Data acquisition from inertia sensors

Two types of sensor devices were used in the study. The accelerometer and gyroscope data were collected from the patient’s index finger, using a high-resolution miniature inertia sensor (LPMS-B2 STD, Omni Instruments, United Kingdom). The motion data was also collected using an iPhone 8 smartphone using Medotemic’s app *Medoclinic*. The phone was placed on top of the patient’s hand as part of the measurement procedure, thereby causing the hand an additional weight of 148 g. The data recorded using the smartphone was sampled at 100 Hz.

### Creation of spectra and tremor indices

Spectrograms were calculated for each channel with 50%-overlapping 8-s windows using the Irregularly Resampled AutoSpectral Analysis method (IRASA). Applying this method, rhythmic components were isolated by dividing the mixed spectra with the resulting fractal spectra (Wen and Liu, 2016). To construct a tremor index for each time window the spectra from individual channels were smoothed with a Gaussian window (with sigma = 1Hz) and the maximum amplitude between 3.5 and 10 Hz was identified. A tremor index for the whole 1-min recording was constructed by taking the median of the 33% of the time windows with the highest tremor indices across time and channels (the algorithm is available as MATLAB code at https://github.com/NRC-Lund/tremordetector). For display purposes, the tremor index was sometimes converted to a decibel scale normalized to the population average of the control group according to

$$I_{d\,B\,\left(C\,T\,R\,L\right)}=10\,\log_{10}\frac{I}{\left\langle I_{C\,T\,R\,L}\right\rangle }.$$

## Results

### Characterization of postural tremor in essential tremor

**FIGURE 1:**
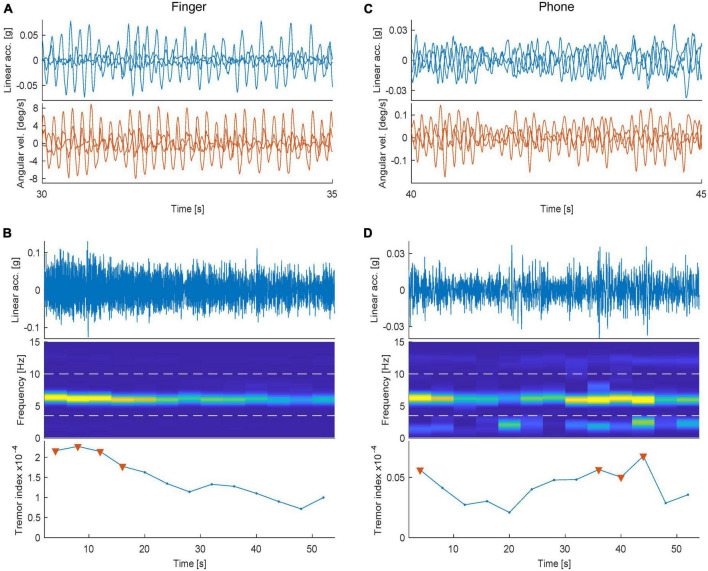
Data processing steps used to create tremor scores. **(A)**, Example raw traces illustrating postural tremor recorded using a high-resolution inertia sensor attached to the index finger. Each line in the two plots illustrates an independently recorded dimension (Top panel: Linear acceleration and Bottom: Angular velocity). **(B)** (Top) Example, one-minute recording of one of the channels shown in panel **(A)**. (Middle) Spectrogram illustrating the relative power spectral density for frequencies <15 Hz, binned in 4s-windows. (Bottom) Calculated tremor index plotted over time [triangles denote the period (top 33%) used to construct a single tremor score]. **(C,D)** The corresponding data sampled from the smartphone (in the same subject recorded directly afterward).

To investigate the feasibility of adaptive DBS based on sensor data from simple self-tests performed in a standardized setting, we initially recorded tremor characteristics using smartphone accelerometry in 17 ET patients during standard neurological evaluation of postural tremor in the clinic (Table 1). The recordings were obtained from an ordinary smartphone (an Apple iPhone 8), being strapped to the dorsum of the tremor-dominant hand. Moreover, to characterize also small digit movements that might potentially not be captured by the smartphone (due to mode/location of attachment, the weight of the device, measurement sensitivity, etc.), similar measurements were performed directly before each smartphone recording using a high-resolution miniature inertia sensor (LPMS-B2 STD, Omni Instruments, United Kingdom) attached to the subjects’ index finger of the tremor-dominant hand. Both sensor types are equipped with 3D linear accelerometers as well as 3D angular velocity sensors, producing six independently sampled data streams (Figures 1A,C). In order to produce a one-dimensional metric that could theoretically be used for closed-loop control, a relatively simple data processing procedure was applied where the dominant tremor frequencies recorded by the 2 × 3 (linear/angular) sensors were used to create two compound tremor indices (data processing steps are summarized in Figures 1B,D).

**FIGURE 2:**
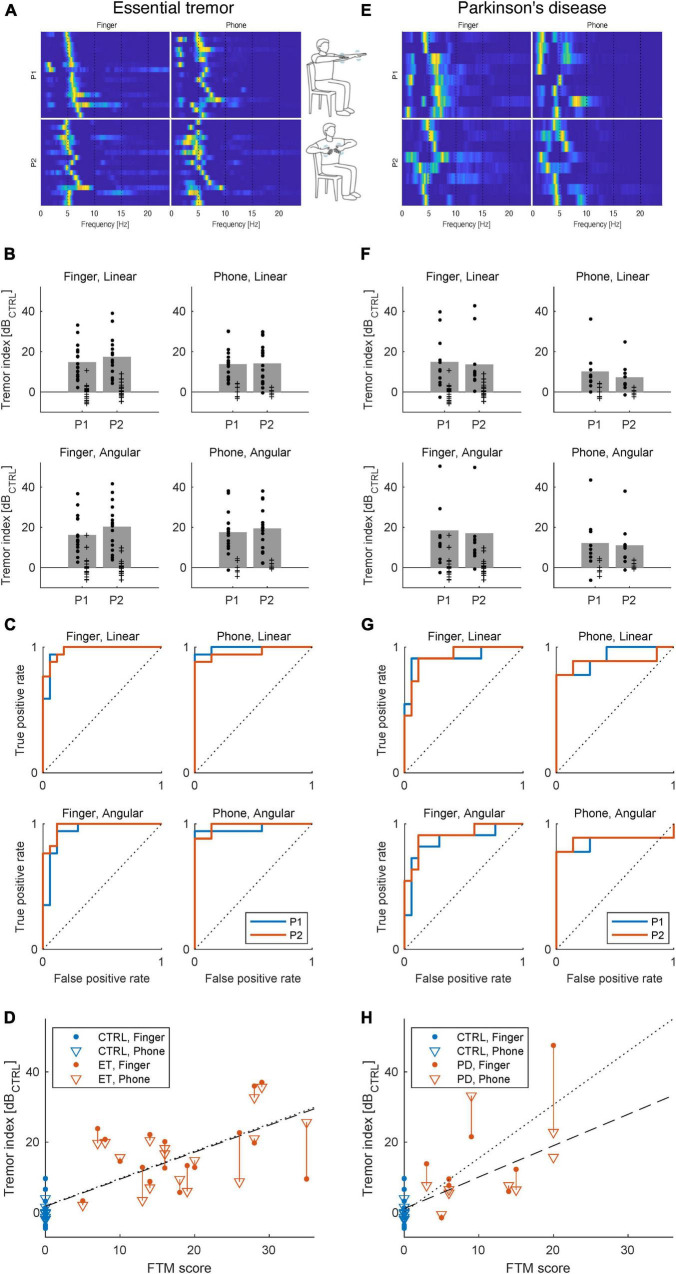
Characterization of postural tremor in ET **(A–D)** and PD **(E–H)** using smartphone or finger-attached sensors. **(A)** Postural tremor recorded using high-resolution inertia sensor attached to the index finger (leftmost column) or smartphone (right). Each line illustrate the relative power spectral density of tremor frequencies in the range 1–20 Hz for one of the 17 ET patients (the 17 patients displayed in each panel were ordered according to each individual’s dominant tremor frequency in Position 1 (P1) using the finger sensor). **(B)** Tremor patterns differ between ET patients and the control group resulting in differences in calculated indices (Rows: linear/angular and Columns: Finger/smartphone sensors). Bars denote means scores above the control group average for the ET patients in the respective conditions and each dot represent one subject (crosses represent control group). **(C)** Receiver operating characteristic curves illustrating classification performance for each data type. **(D)** Correlation of tremor index to clinical FTM scores. The dotted and dashed lines are the least-squared fits to the Finger and Phone data, respectively. **(E–H)** The corresponding data for PD patients. Note that for both ET and PD patients, linear and angular data obtained with either of the two sensors (finger-attached/smartphone) both resulted in relatively high classification performance (linear acceleration for finger and smartphone, respectively, ET AUC: P1/P2:0.969/0.976 and 0.992/0.958 and angular velocity for finger and smartphone, respectively, ET AUC P1/P2:0.938/0.976 and 0.966/0.983). and for PD (linear acceleration for finger and smartphone, respectively, PD AUC: P1/P2:0.918/0.925 and 0.921/0.889 and angular velocity for finger and smartphone, respectively, PD AUC P1/P2:0.886/0.909 and 0.857/0.873). Illustrations of tremor positions adapted from Isaacson et al. (2020). For panels **(B,D,F,H)** tremor index values were normalized to the population average of the control group and logarithmized for easier comparison.

In all 17 subjects, key characteristics of ET postural tremor was detected in at least one of the two positions (P1/P2) tested, although to a varying degree. Especially, when analyzing the tremor frequency components, a distinct peak at 5-8 Hz was found in all ET subjects (for P1, mean ± SD = 6.0 ± 1.0; Figure 2A; see also Supplementary Figure 1). Hence the individual 1D tremor indices extracted from the recorded data, resulted in reliable identification of ET tremor features, as evident when comparing the indices of the ET patients to the corresponding indices for the control group (where physiological tremor instead was the dominating movement pattern; Figure 2B). For this purpose, linear acceleration and angular velocity appeared to provide equally useful movement descriptors (Figure 2C). Perhaps more surprisingly, the separation between the ET group and controls using the obtained tremor indices was found to be very similar for the smartphone and the finger sensor (Figure 2C). Indeed, plotting the tremor scores from the smartphone recordings against the scores from the finger-attached sensor it was evident that, even on an individual level, there was a great correspondence between the two sensors (Figure 2D). Importantly, the calculated tremor indices were also found to correlate well with clinical tremor assessments using the Fahn-Tolosa-Marine (FTM) rating scale (Figure 2D; Pearson correlation of FTM scores against tremor scores, r^2^ = 0.58/0.59, for finger/smartphone sensors, respectively).

### Characterization of postural tremor in Parkinson’s disease

Because our findings in ET patients suggest that data from smartphone accelerometry sensors can provide detailed descriptors of tremor in this patient group, we next wanted to evaluate the corresponding procedures in PD patients. As alluded to in the introduction it is likely that certain aspects of a future closed-loop DBS design will differ between ET and PD patients. Nonetheless, for the current purpose of evaluating sensor characteristics it is clearly highly relevant to test both patient groups under similar conditions, since tremor characteristics are known to differ somewhat between ET and PD. Thus, to investigate if the same procedures could be used to characterize tremor also in PD patients, we next performed the same type of tremor recordings in nine PD patients (Table 1). Although, less pronounced than in the ET group, postural tremor was detected in the majority of PD patients in at least one of the two positions tested. The postural tremor detected shared several features of ET tremor and had broadly similar main frequency components (for P1, mean ± SD = 6.3 ± 1.3; Figure 2E; see also Supplementary Figure 2). Interestingly, even though postural tremor was less pronounced in PD compared to ET, it was evident that based on the calculated tremor indices PD patients were clearly separable from the control group (Figure 2F). Just as for the ET group, linear acceleration and angular velocity sensors both provided equally useful movement descriptors (Figure 2G) and plotting the tremor scores from the smartphone recordings against the scores from the finger-attached sensor, we could again confirm a high correspondence between the two sensors and a clear correlation to clinical scores (Figure 2H; Pearson correlation of FTM scores against tremor scores, *r*^2^ = 0.62/0.45, for finger/smartphone sensors, respectively). Taken together, the testing procedure used appeared to result in relatively reliable estimates of postural tremor, also in PD.

### Direct comparison of finger-attached sensor to smartphone accelerometry

**FIGURE 3:**
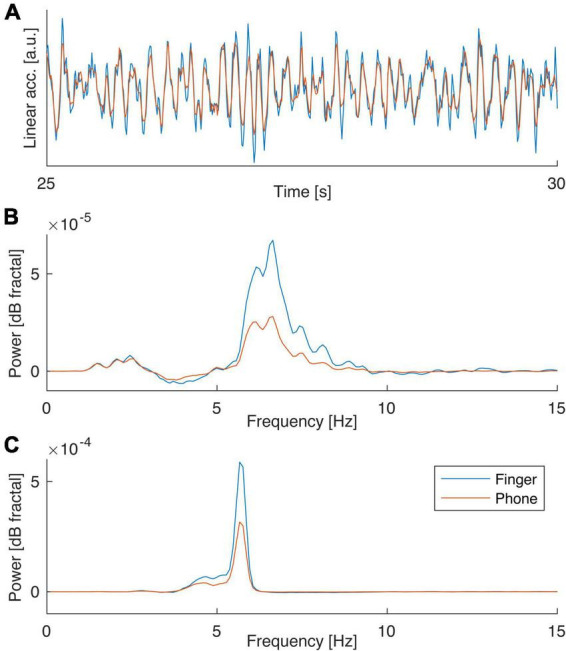
Direct comparison of finger-attached sensor to smartphone accelerometry. **(A)** Example recordings from finger/smartphone sensors performed in parallel in an ET patient. **(B)** Power spectral density of postural tremor data obtained in the two positions (P1/P2) with the two sensors. Note the great resemblance in spectral contents suggesting the main difference is a difference in signal-to-noise (the ratio in peak value over background for finger to phone sensor were 2.4 and 1.9 respectively for P1 and P2).

Intriguingly, our data showed very similar tremor characteristics when using either the smartphone strapped to the hand or the high-resolution inertia sensor attached to the index finger. In a control experiment, we therefore directly compare the properties of these two sensors recording tremor features in an ET patient using both sensors in a parallel arrangement measuring from the dorsum and the index finger of the same hand. The experiment demonstrated that the sensors indeed display very similar measurements in spite of the differences in location of attachment, sensor geometry in relation to joints etc. An example recording from the linear accelerometers in the two sensors is shown in Figure 3A.

Notably, when summarizing the power spectral density of the tremor characteristics recorded over the entire 1-minute period for the two positions P1 and P2 it was evident that the spectral properties of the signals recorded in the two sensors were very similar. In fact, the only apparent difference was a slightly higher signal-to-noise as suggested by a significantly higher peak above the noise background in the two spectra for the finger sensor compared to the phone (Figures 3B,C; *p* = 0.0017 for P1 and *p* = 0.0034 P2, Wilcoxon signed rank test).

## Discussion

Based on the current findings, we suggest that a self-testing procedure for postural tremor using smartphone inertia sensors could in the future be used to greatly facilitate on-demand stimulation adjustments in neuromodulation therapies. The procedure described herein is simple enough for patients to perform independently at home, several times a day if needed, with the aim of adapting therapies to the changing needs of the patient, for example via adjustments of DBS stimulation patterns (see also Fuentes et al., 2009; Santana et al., 2014; Petersson et al., 2020), or possibly for changes in medication regimens. Using the standardized testing conditions described, smartphone accelerometry proved according to our results in many cases to be equally sensitive and reliable as high-resolution measurements using a finger-attached inertia sensor (which in this context can be regarded as a good approximation of the ground-truth with respect to the tremor patters displayed). The procedures were here evaluated in both ET and PD patients. To allow for comparisons, the exact same procedures were used for ET and PD patients. It can be noted however, that a standardized testing design including an assessment of resting tremor, could potentially further improve the characterization of tremor features, perhaps in particular for PD tremor (Vial et al., 2019). In the current experiments, a smartphone was used since this is a widely used consumer product containing inertia sensors. It should be noted that the test design proposed, using smartphones that have a substantially larger weight than high-resolution finger sensors, is mainly applicable for central tremors (such as essential and Parkinsonian) and should be used with some caution for measurements of peripheral tremors, such as exaggerated physiological tremor, given that previous studies have demonstrated an effect of external limb loading on tremor frequency in peripheral tremors (as opposed to central; Hallett, 1998). In recent years, smartwatches have become more popular and could therefore offer a more convenient alternative (López-Blanco et al., 2019). It needs to be cautioned however, that motion patterns recorded at the wrist may differ considerably from the tremor patterns recorded in the current study. Overall, the rapid expansion of new sensor technologies has opened the door for new methods to optimize treatments in movement disorders. In this context, our current study represents an example of how to incorporate existing sensor technologies to guide therapies and improve the lives of patients afflicted with tremor related diseases.

## Data availability statement

The raw data supporting the conclusions of this article will be made available by the authors, without undue reservation.

## Ethics statement

The studies involving human participants were reviewed and approved by The Swedish Ethical Review Authority. The patients/participants provided their written informed consent to participate in this study.

## Author contributions

GS, PH, AJ, and PP designed the experiments. GS and SU performed the experiments. PH analyzed the data. PP wrote the manuscript draft. All authors took part in revising the manuscript draft and have approved the final version.
