# Supplementary material for: Tremor evaluation using smartphone accelerometry in standardized settings
Source: Front Neurosci. 2022 Aug 1;16:861668. doi: 10.3389/fnins.2022.861668 (PMC9376601; doi:10.3389/fnins.2022.861668)
Supplement: Supplementary file 1 [file Data_Sheet_1.pdf]

Supplementary Material

1. Supplementary Figures

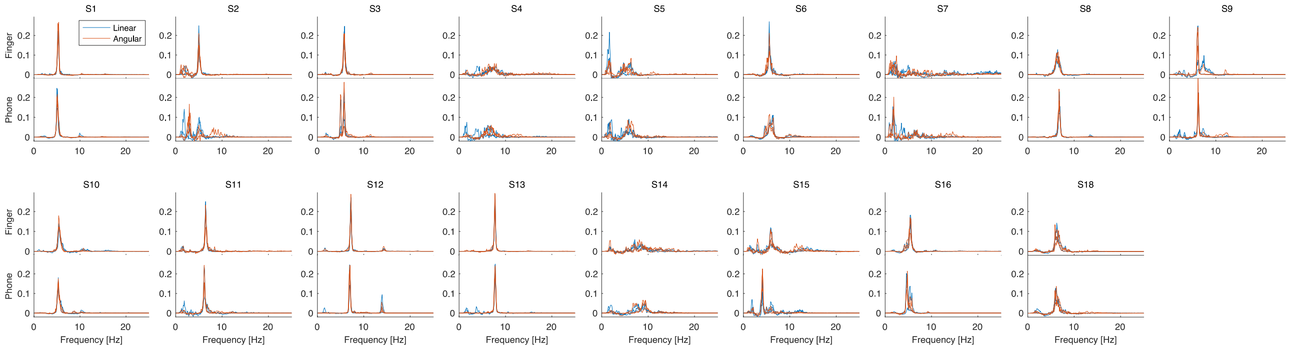

Supplementary Figure 1. Power spectral density of postural tremor in all ET patients plotted for recording condition position P1.

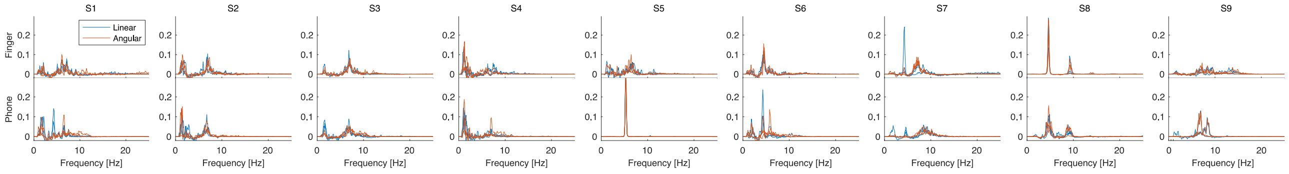

Supplementary Figure 2. Power spectral density of postural tremor in all PD patients plotted for recording condition position P1.
